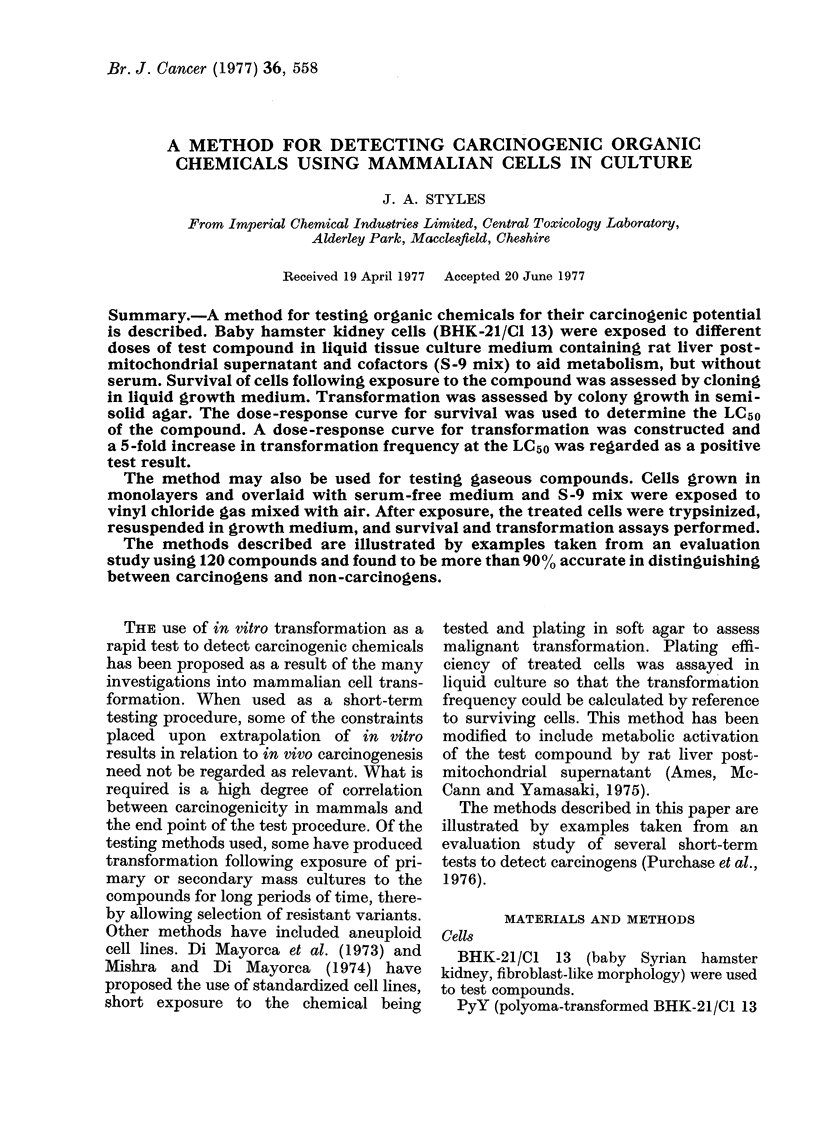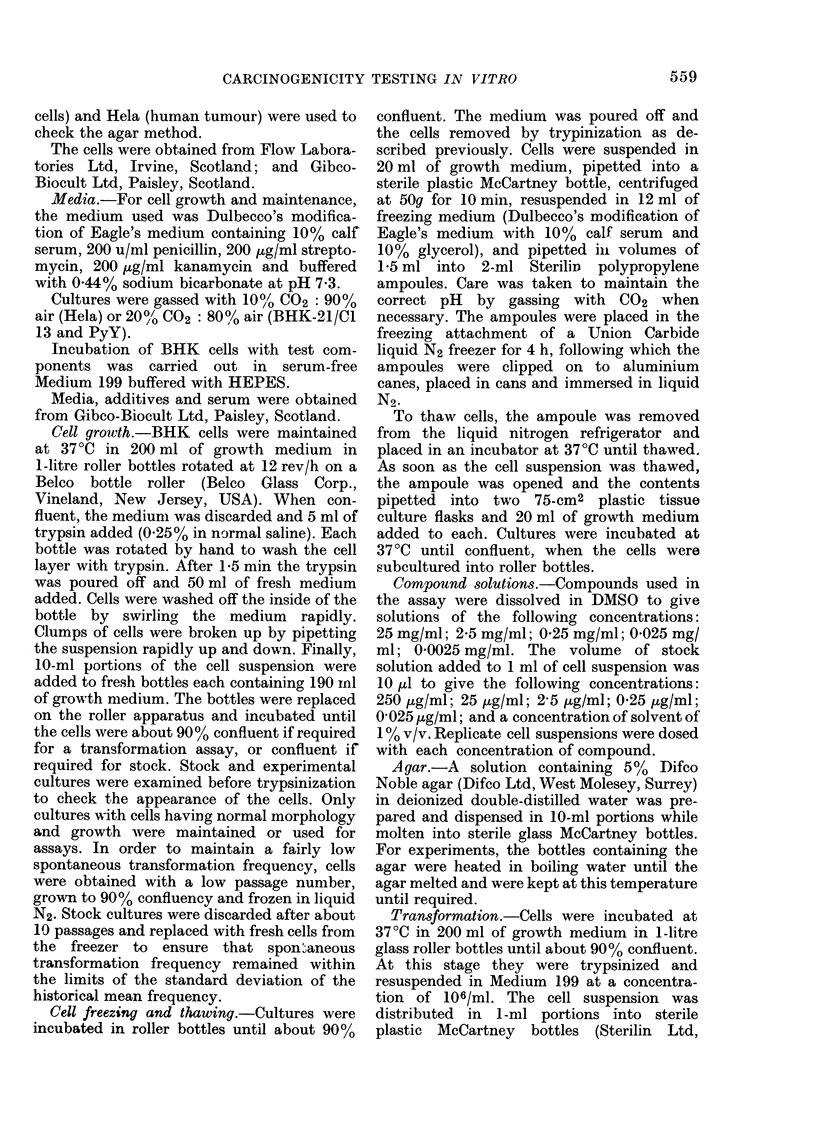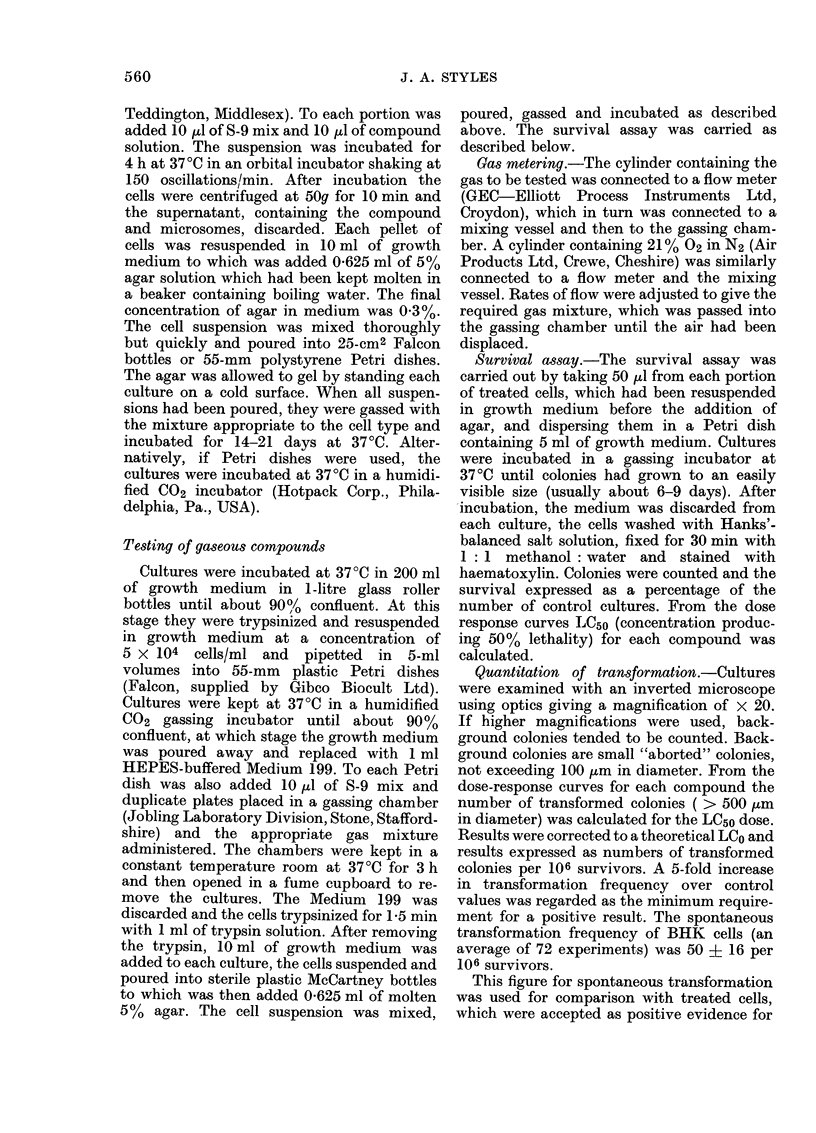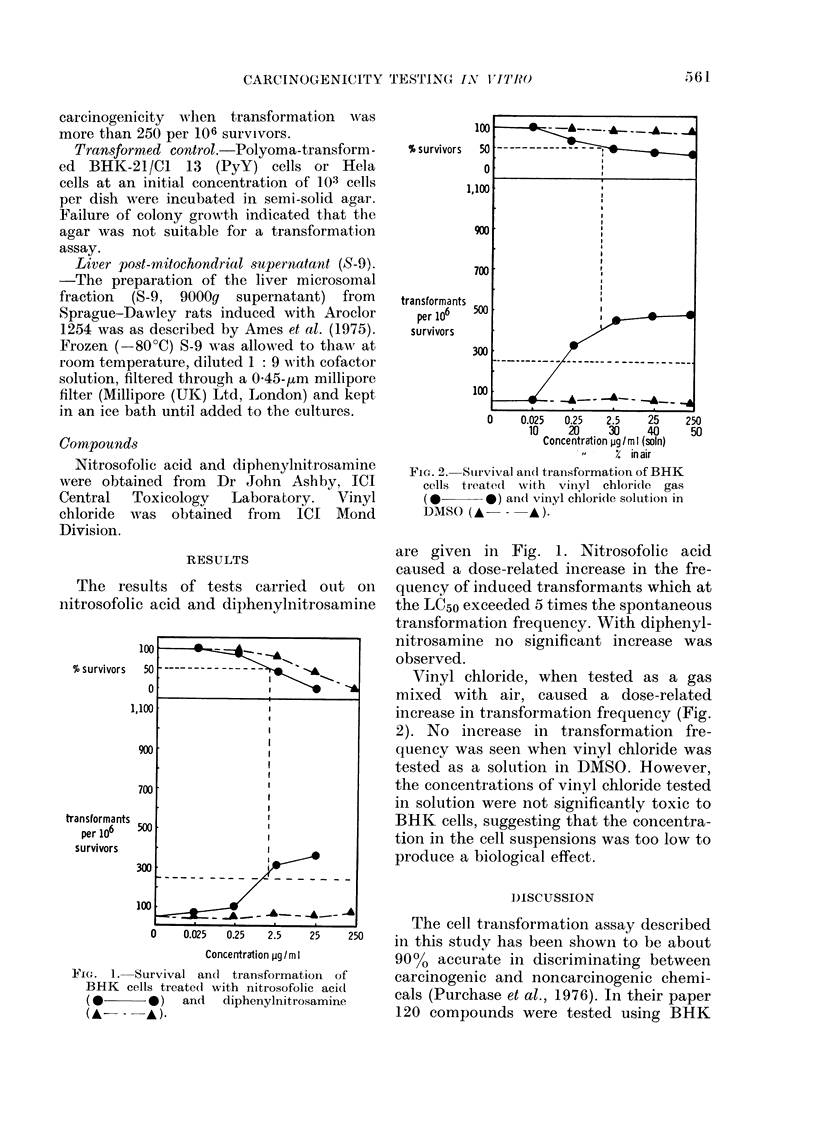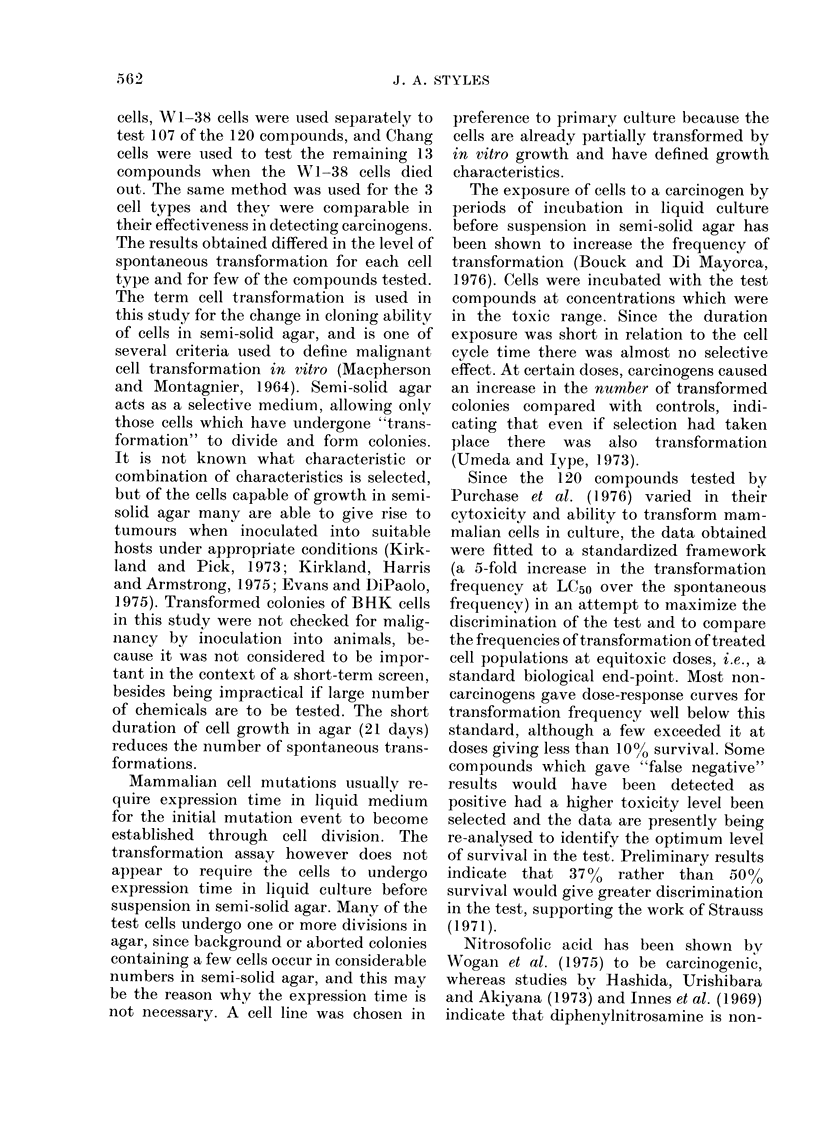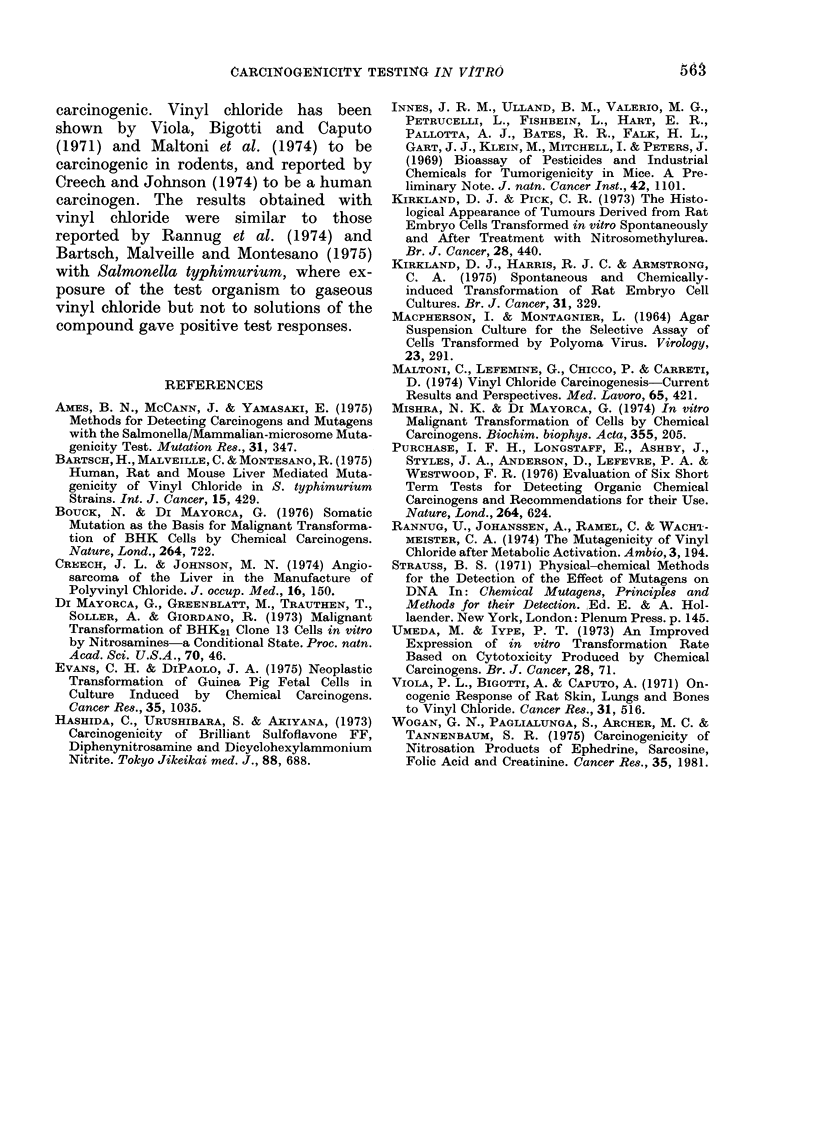# A method for detecting carcinogenic organic chemicals using mammalian cells in culture.

**DOI:** 10.1038/bjc.1977.231

**Published:** 1977-11

**Authors:** J. A. Styles

## Abstract

A method for testing organic chemicals for their carcinogenic potential is described. Baby hamster kidney cells (BHK-21/C1 13) were exposed to different doses of test compound in liquid tissue culture medium containing rat liver post-mitochondrial supernatant and cofactors (S-9 mix) to aid metabolism, but without serum. Survival of cells following exposure to the compound was assessed by cloning in liquid growth medium. Transformation was assessed by colony growth in semi-solid agar. The dose-response curve for survival was used to determine the LC50 of the compound. A dose-response curve for transformation was constructed and a 5-fold increase in transformation frequency at the LC50 was regarded as a positive test result. The method may also be used for testing gaseous compounds. Cells grown in monolayers and overlaid with serum-free medium and S-9 mix were exposed to vinyl chloride gas mixed with air. After exposure, the treated cells were trypsinized, resuspended in growth medium, and survival and transformation assays performed. The methods described are illustrated by examples taken from an evaluation study using 120 compounds and found to be more than 90% accurate in distinguishing between carcinogens and non-carcinogens.


*Br. J. Cancer* (1977) **36**, 558

### A METHOD FOR DETECTING CARCINOGENIC ORGANIC CHEMICALS USING MAMMALIAN CELLS IN CULTURE

J. A. STYLES

*From Imperial Chemical Industries Limited, Central Toxicology Laboratory, Alderley Park, Macclesfield, Cheshire*

Received 19 April 1977 Accepted 20 June 1977

**Summary.—A method for testing organic chemicals for their carcinogenic potential is described. Baby hamster kidney cells (BHK-21/Cl 13) were exposed to different doses of test compound in liquid tissue culture medium containing rat liver post-mitochondrial supernatant and cofactors (S-9 mix) to aid metabolism, but without serum. Survival of cells following exposure to the compound was assessed by cloning in liquid growth medium. Transformation was assessed by colony growth in semi-solid agar. The dose-response curve for survival was used to determine the $LC_{50}$ of the compound. A dose-response curve for transformation was constructed and a 5-fold increase in transformation frequency at the $LC_{50}$ was regarded as a positive test result.**

**The method may also be used for testing gaseous compounds. Cells grown in monolayers and overlaid with serum-free medium and S-9 mix were exposed to vinyl chloride gas mixed with air. After exposure, the treated cells were trypsinized, resuspended in growth medium, and survival and transformation assays performed.**

**The methods described are illustrated by examples taken from an evaluation study using 120 compounds and found to be more than 90% accurate in distinguishing between carcinogens and non-carcinogens.**

THE use of *in vitro* transformation as a rapid test to detect carcinogenic chemicals has been proposed as a result of the many investigations into mammalian cell transformation. When used as a short-term testing procedure, some of the constraints placed upon extrapolation of *in vitro* results in relation to *in vivo* carcinogenesis need not be regarded as relevant. What is required is a high degree of correlation between carcinogenicity in mammals and the end point of the test procedure. Of the testing methods used, some have produced transformation following exposure of primary or secondary mass cultures to the compounds for long periods of time, thereby allowing selection of resistant variants. Other methods have included aneuploid cell lines. Di Mayorca *et al.* (1973) and Mishra and Di Mayorca (1974) have proposed the use of standardized cell lines, short exposure to the chemical being tested and plating in soft agar to assess malignant transformation. Plating efficiency of treated cells was assayed in liquid culture so that the transformation frequency could be calculated by reference to surviving cells. This method has been modified to include metabolic activation of the test compound by rat liver post-mitochondrial supernatant (Ames, McCann and Yamasaki, 1975).

The methods described in this paper are illustrated by examples taken from an evaluation study of several short-term tests to detect carcinogens (Purchase *et al.*, 1976).

### MATERIALS AND METHODS

### *Cells*

BHK-21/Cl 13 (baby Syrian hamster kidney, fibroblast-like morphology) were used to test compounds.

PyY (polyoma-transformed BHK-21/Cl 13

cells) and Hela (human tumour) were used to check the agar method.

The cells were obtained from Flow Laboratories Ltd, Irvine, Scotland; and Gibco-Biocult Ltd, Paisley, Scotland.

*Media*.—For cell growth and maintenance, the medium used was Dulbecco's modification of Eagle's medium containing 10% calf serum, 200 u/ml penicillin, 200 $\mu$g/ml streptomycin, 200 $\mu$g/ml kanamycin and buffered with 0·44% sodium bicarbonate at pH 7·3.

Cultures were gassed with 10% $CO_2$ : 90% air (Hela) or 20% $CO_2$ : 80% air (BHK-21/Cl 13 and PyY).

Incubation of BHK cells with test components was carried out in serum-free Medium 199 buffered with HEPES.

Media, additives and serum were obtained from Gibco-Biocult Ltd, Paisley, Scotland.

*Cell growth*.—BHK cells were maintained at 37°C in 200 ml of growth medium in 1-litre roller bottles rotated at 12 rev/h on a Belco bottle roller (Belco Glass Corp., Vineland, New Jersey, USA). When confluent, the medium was discarded and 5 ml of trypsin added (0·25% in normal saline). Each bottle was rotated by hand to wash the cell layer with trypsin. After 1·5 min the trypsin was poured off and 50 ml of fresh medium added. Cells were washed off the inside of the bottle by swirling the medium rapidly. Clumps of cells were broken up by pipetting the suspension rapidly up and down. Finally, 10-ml portions of the cell suspension were added to fresh bottles each containing 190 ml of growth medium. The bottles were replaced on the roller apparatus and incubated until the cells were about 90% confluent if required for a transformation assay, or confluent if required for stock. Stock and experimental cultures were examined before trypsinization to check the appearance of the cells. Only cultures with cells having normal morphology and growth were maintained or used for assays. In order to maintain a fairly low spontaneous transformation frequency, cells were obtained with a low passage number, grown to 90% confluency and frozen in liquid $N_2$. Stock cultures were discarded after about 10 passages and replaced with fresh cells from the freezer to ensure that spontaneous transformation frequency remained within the limits of the standard deviation of the historical mean frequency.

*Cell freezing and thawing*.—Cultures were incubated in roller bottles until about 90% confluent. The medium was poured off and the cells removed by trypinization as described previously. Cells were suspended in 20 ml of growth medium, pipetted into a sterile plastic McCartney bottle, centrifuged at 50*g* for 10 min, resuspended in 12 ml of freezing medium (Dulbecco's modification of Eagle's medium with 10% calf serum and 10% glycerol), and pipetted in volumes of 1·5 ml into 2-ml Sterilin polypropylene ampoules. Care was taken to maintain the correct pH by gassing with $CO_2$ when necessary. The ampoules were placed in the freezing attachment of a Union Carbide liquid $N_2$ freezer for 4 h, following which the ampoules were clipped on to aluminium canes, placed in cans and immersed in liquid $N_2$.

To thaw cells, the ampoule was removed from the liquid nitrogen refrigerator and placed in an incubator at 37°C until thawed. As soon as the cell suspension was thawed, the ampoule was opened and the contents pipetted into two 75-cm$^2$ plastic tissue culture flasks and 20 ml of growth medium added to each. Cultures were incubated at 37°C until confluent, when the cells were subcultured into roller bottles.

*Compound solutions*.—Compounds used in the assay were dissolved in DMSO to give solutions of the following concentrations: 25 mg/ml; 2·5 mg/ml; 0·25 mg/ml; 0·025 mg/ml; 0·0025 mg/ml. The volume of stock solution added to 1 ml of cell suspension was 10 $\mu$l to give the following concentrations: 250 $\mu$g/ml; 25 $\mu$g/ml; 2·5 $\mu$g/ml; 0·25 $\mu$g/ml; 0·025 $\mu$g/ml; and a concentration of solvent of 1% v/v. Replicate cell suspensions were dosed with each concentration of compound.

*Agar*.—A solution containing 5% Difco Noble agar (Difco Ltd, West Molesey, Surrey) in deionized double-distilled water was prepared and dispensed in 10-ml portions while molten into sterile glass McCartney bottles. For experiments, the bottles containing the agar were heated in boiling water until the agar melted and were kept at this temperature until required.

*Transformation*.—Cells were incubated at 37°C in 200 ml of growth medium in 1-litre glass roller bottles until about 90% confluent. At this stage they were trypsinized and resuspended in Medium 199 at a concentration of $10^6$/ml. The cell suspension was distributed in 1-ml portions into sterile plastic McCartney bottles (Sterilin Ltd,

Teddington, Middlesex). To each portion was added 10 $\mu$l of S-9 mix and 10 $\mu$l of compound solution. The suspension was incubated for 4 h at 37 °C in an orbital incubator shaking at 150 oscillations/min. After incubation the cells were centrifuged at 50*g* for 10 min and the supernatant, containing the compound and microsomes, discarded. Each pellet of cells was resuspended in 10 ml of growth medium to which was added 0·625 ml of 5% agar solution which had been kept molten in a beaker containing boiling water. The final concentration of agar in medium was 0·3%. The cell suspension was mixed thoroughly but quickly and poured into 25-cm$^2$ Falcon bottles or 55-mm polystyrene Petri dishes. The agar was allowed to gel by standing each culture on a cold surface. When all suspensions had been poured, they were gassed with the mixture appropriate to the cell type and incubated for 14–21 days at 37°C. Alternatively, if Petri dishes were used, the cultures were incubated at 37 °C in a humidified $CO_2$ incubator (Hotpack Corp., Philadelphia, Pa., USA).

*Testing of gaseous compounds*

Cultures were incubated at 37 °C in 200 ml of growth medium in 1-litre glass roller bottles until about 90% confluent. At this stage they were trypsinized and resuspended in growth medium at a concentration of $5 \times 10^4$ cells/ml and pipetted in 5-ml volumes into 55-mm plastic Petri dishes (Falcon, supplied by Gibco Biocult Ltd). Cultures were kept at 37 °C in a humidified $CO_2$ gassing incubator until about 90% confluent, at which stage the growth medium was poured away and replaced with 1 ml HEPES-buffered Medium 199. To each Petri dish was also added 10 $\mu$l of S-9 mix and duplicate plates placed in a gassing chamber (Jobling Laboratory Division, Stone, Staffordshire) and the appropriate gas mixture administered. The chambers were kept in a constant temperature room at 37 °C for 3 h and then opened in a fume cupboard to remove the cultures. The Medium 199 was discarded and the cells trypsinized for 1·5 min with 1 ml of trypsin solution. After removing the trypsin, 10 ml of growth medium was added to each culture, the cells suspended and poured into sterile plastic McCartney bottles to which was then added 0·625 ml of molten 5% agar. The cell suspension was mixed, poured, gassed and incubated as described above. The survival assay was carried as described below.

*Gas metering.*—The cylinder containing the gas to be tested was connected to a flow meter (GEC—Elliott Process Instruments Ltd, Croydon), which in turn was connected to a mixing vessel and then to the gassing chamber. A cylinder containing 21% $O_2$ in $N_2$ (Air Products Ltd, Crewe, Cheshire) was similarly connected to a flow meter and the mixing vessel. Rates of flow were adjusted to give the required gas mixture, which was passed into the gassing chamber until the air had been displaced.

*Survival assay.*—The survival assay was carried out by taking 50 $\mu$l from each portion of treated cells, which had been resuspended in growth medium before the addition of agar, and dispersing them in a Petri dish containing 5 ml of growth medium. Cultures were incubated in a gassing incubator at 37 °C until colonies had grown to an easily visible size (usually about 6–9 days). After incubation, the medium was discarded from each culture, the cells washed with Hanks'-balanced salt solution, fixed for 30 min with 1 : 1 methanol : water and stained with haematoxylin. Colonies were counted and the survival expressed as a percentage of the number of control cultures. From the dose response curves $LC_{50}$ (concentration producing 50% lethality) for each compound was calculated.

*Quantitation of transformation.*—Cultures were examined with an inverted microscope using optics giving a magnification of $\times$ 20. If higher magnifications were used, background colonies tended to be counted. Background colonies are small "aborted" colonies, not exceeding 100 $\mu$m in diameter. From the dose-response curves for each compound the number of transformed colonies ( $> 500$ $\mu$m in diameter) was calculated for the $LC_{50}$ dose. Results were corrected to a theoretical $LC_0$ and results expressed as numbers of transformed colonies per $10^6$ survivors. A 5-fold increase in transformation frequency over control values was regarded as the minimum requirement for a positive result. The spontaneous transformation frequency of BHK cells (an average of 72 experiments) was $50 \pm 16$ per $10^6$ survivors.

This figure for spontaneous transformation was used for comparison with treated cells, which were accepted as positive evidence for carcinogenicity when transformation was more than 250 per $10^6$ survivors.

*Transformed control.*—Polyoma-transformed BHK-21/Cl 13 (PyY) cells or Hela cells at an initial concentration of $10^3$ cells per dish were incubated in semi-solid agar. Failure of colony growth indicated that the agar was not suitable for a transformation assay.

*Liver post-mitochondrial supernatant* (*S*-9).—The preparation of the liver microsomal fraction (S-9, 9000*g* supernatant) from Sprague–Dawley rats induced with Aroclor 1254 was as described by Ames *et al.* (1975). Frozen (−80°C) S-9 was allowed to thaw at room temperature, diluted 1 : 9 with cofactor solution, filtered through a 0·45-μm millipore filter (Millipore (UK) Ltd, London) and kept in an ice bath until added to the cultures.

*Compounds*

Nitrosofolic acid and diphenylnitrosamine were obtained from Dr John Ashby, ICI Central Toxicology Laboratory. Vinyl chloride was obtained from ICI Mond Division.

RESULTS

The results of tests carried out on nitrosofolic acid and diphenylnitrosamine

FIG. 1.—Survival and transformation of BHK cells treated with nitrosofolic acid (●———●) and diphenylnitrosamine (▲— - —▲).

FIG. 2.—Survival and transformation of BHK cells treated with vinyl chloride gas (●———●) and vinyl chloride solution in DMSO (▲— - —▲).

are given in Fig. 1. Nitrosofolic acid caused a dose-related increase in the frequency of induced transformants which at the $LC_{50}$ exceeded 5 times the spontaneous transformation frequency. With diphenylnitrosamine no significant increase was observed.

Vinyl chloride, when tested as a gas mixed with air, caused a dose-related increase in transformation frequency (Fig. 2). No increase in transformation frequency was seen when vinyl chloride was tested as a solution in DMSO. However, the concentrations of vinyl chloride tested in solution were not significantly toxic to BHK cells, suggesting that the concentration in the cell suspensions was too low to produce a biological effect.

DISCUSSION

The cell transformation assay described in this study has been shown to be about 90% accurate in discriminating between carcinogenic and noncarcinogenic chemicals (Purchase *et al.*, 1976). In their paper 120 compounds were tested using BHK

cells, W1–38 cells were used separately to test 107 of the 120 compounds, and Chang cells were used to test the remaining 13 compounds when the W1–38 cells died out. The same method was used for the 3 cell types and they were comparable in their effectiveness in detecting carcinogens. The results obtained differed in the level of spontaneous transformation for each cell type and for few of the compounds tested. The term cell transformation is used in this study for the change in cloning ability of cells in semi-solid agar, and is one of several criteria used to define malignant cell transformation *in vitro* (Macpherson and Montagnier, 1964). Semi-solid agar acts as a selective medium, allowing only those cells which have undergone "transformation" to divide and form colonies. It is not known what characteristic or combination of characteristics is selected, but of the cells capable of growth in semi-solid agar many are able to give rise to tumours when inoculated into suitable hosts under appropriate conditions (Kirkland and Pick, 1973; Kirkland, Harris and Armstrong, 1975; Evans and DiPaolo, 1975). Transformed colonies of BHK cells in this study were not checked for malignancy by inoculation into animals, because it was not considered to be important in the context of a short-term screen, besides being impractical if large number of chemicals are to be tested. The short duration of cell growth in agar (21 days) reduces the number of spontaneous transformations.

Mammalian cell mutations usually require expression time in liquid medium for the initial mutation event to become established through cell division. The transformation assay however does not appear to require the cells to undergo expression time in liquid culture before suspension in semi-solid agar. Many of the test cells undergo one or more divisions in agar, since background or aborted colonies containing a few cells occur in considerable numbers in semi-solid agar, and this may be the reason why the expression time is not necessary. A cell line was chosen in preference to primary culture because the cells are already partially transformed by *in vitro* growth and have defined growth characteristics.

The exposure of cells to a carcinogen by periods of incubation in liquid culture before suspension in semi-solid agar has been shown to increase the frequency of transformation (Bouck and Di Mayorca, 1976). Cells were incubated with the test compounds at concentrations which were in the toxic range. Since the duration exposure was short in relation to the cell cycle time there was almost no selective effect. At certain doses, carcinogens caused an increase in the *number* of transformed colonies compared with controls, indicating that even if selection had taken place there was also transformation (Umeda and Iype, 1973).

Since the 120 compounds tested by Purchase *et al.* (1976) varied in their cytoxicity and ability to transform mammalian cells in culture, the data obtained were fitted to a standardized framework (a 5-fold increase in the transformation frequency at $LC_{50}$ over the spontaneous frequency) in an attempt to maximize the discrimination of the test and to compare the frequencies of transformation of treated cell populations at equitoxic doses, *i.e.*, a standard biological end-point. Most non-carcinogens gave dose-response curves for transformation frequency well below this standard, although a few exceeded it at doses giving less than 10% survival. Some compounds which gave "false negative" results would have been detected as positive had a higher toxicity level been selected and the data are presently being re-analysed to identify the optimum level of survival in the test. Preliminary results indicate that 37% rather than 50% survival would give greater discrimination in the test, supporting the work of Strauss (1971).

Nitrosofolic acid has been shown by Wogan *et al.* (1975) to be carcinogenic, whereas studies by Hashida, Urishibara and Akiyana (1973) and Innes *et al.* (1969) indicate that diphenylnitrosamine is noncarcinogenic. Vinyl chloride has been shown by Viola, Bigotti and Caputo (1971) and Maltoni *et al.* (1974) to be carcinogenic in rodents, and reported by Creech and Johnson (1974) to be a human carcinogen. The results obtained with vinyl chloride were similar to those reported by Rannug *et al.* (1974) and Bartsch, Malveille and Montesano (1975) with *Salmonella typhimurium*, where exposure of the test organism to gaseous vinyl chloride but not to solutions of the compound gave positive test responses.

### REFERENCES

AMES, B. N., McCANN, J. & YAMASAKI, E. (1975) Methods for Detecting Carcinogens and Mutagens with the Salmonella/Mammalian-microsome Mutagenicity Test. *Mutation Res.*, **31**, 347.

BARTSCH, H., MALVEILLE, C. & MONTESANO, R. (1975) Human, Rat and Mouse Liver Mediated Mutagenicity of Vinyl Chloride in *S. typhimurium* Strains. *Int. J. Cancer*, **15**, 429.

BOUCK, N. & DI MAYORCA, G. (1976) Somatic Mutation as the Basis for Malignant Transformation of BHK Cells by Chemical Carcinogens. *Nature, Lond.*, **264**, 722.

CREECH, J. L. & JOHNSON, M. N. (1974) Angiosarcoma of the Liver in the Manufacture of Polyvinyl Chloride. *J. occup. Med.*, **16**, 150.

DI MAYORCA, G., GREENBLATT, M., TRAUTHEN, T., SOLLER, A. & GIORDANO, R. (1973) Malignant Transformation of $BHK_{21}$ Clone 13 Cells *in vitro* by Nitrosamines—a Conditional State. *Proc. natn. Acad. Sci. U.S.A.*, **70**, 46.

EVANS, C. H. & DIPAOLO, J. A. (1975) Neoplastic Transformation of Guinea Pig Fetal Cells in Culture Induced by Chemical Carcinogens. *Cancer Res.*, **35**, 1035.

HASHIDA, C., URUSHIBARA, S. & AKIYANA, (1973) Carcinogenicity of Brilliant Sulfoflavone FF, Diphenynitrosamine and Dicyclohexylammonium Nitrite. *Tokyo Jikeikai med. J.*, **88**, 688.

INNES, J. R. M., ULLAND, B. M., VALERIO, M. G., PETRUCELLI, L., FISHBEIN, L., HART, E. R., PALLOTTA, A. J., BATES, R. R., FALK, H. L., GART, J. J., KLEIN, M., MITCHELL, I. & PETERS, J. (1969) Bioassay of Pesticides and Industrial Chemicals for Tumorigenicity in Mice. A Preliminary Note. *J. natn. Cancer Inst.*, **42**, 1101.

KIRKLAND, D. J. & PICK, C. R. (1973) The Histological Appearance of Tumours Derived from Rat Embryo Cells Transformed *in vitro* Spontaneously and After Treatment with Nitrosomethylurea. *Br. J. Cancer*, **28**, 440.

KIRKLAND, D. J., HARRIS, R. J. C. & ARMSTRONG, C. A. (1975) Spontaneous and Chemically-induced Transformation of Rat Embryo Cell Cultures. *Br. J. Cancer*, **31**, 329.

MACPHERSON, I. & MONTAGNIER, L. (1964) Agar Suspension Culture for the Selective Assay of Cells Transformed by Polyoma Virus. *Virology*, **23**, 291.

MALTONI, C., LEFEMINE, G., CHICCO, P. & CARRETI, D. (1974) Vinyl Chloride Carcinogenesis—Current Results and Perspectives. *Med. Lavoro*, **65**, 421.

MISHRA, N. K. & DI MAYORCA, G. (1974) *In vitro* Malignant Transformation of Cells by Chemical Carcinogens. *Biochim. biophys. Acta*, **355**, 205.

PURCHASE, I. F. H., LONGSTAFF, E., ASHBY, J., STYLES, J. A., ANDERSON, D., LEFEVRE, P. A. & WESTWOOD, F. R. (1976) Evaluation of Six Short Term Tests for Detecting Organic Chemical Carcinogens and Recommendations for their Use. *Nature, Lond.*, **264**, 624.

RANNUG, U., JOHANSSEN, A., RAMEL, C. & WACHTMEISTER, C. A. (1974) The Mutagenicity of Vinyl Chloride after Metabolic Activation. *Ambio*, **3**, 194.

STRAUSS, B. S. (1971) Physical–chemical Methods for the Detection of the Effect of Mutagens on DNA In: *Chemical Mutagens, Principles and Methods for their Detection.* Ed. E. & A. Hollaender. New York, London: Plenum Press. p. 145.

UMEDA, M. & IYPE, P. T. (1973) An Improved Expression of *in vitro* Transformation Rate Based on Cytotoxicity Produced by Chemical Carcinogens. *Br. J. Cancer*, **28**, 71.

VIOLA, P. L., BIGOTTI, A. & CAPUTO, A. (1971) Oncogenic Response of Rat Skin, Lungs and Bones to Vinyl Chloride. *Cancer Res.*, **31**, 516.

WOGAN, G. N., PAGLIALUNGA, S., ARCHER, M. C. & TANNENBAUM, S. R. (1975) Carcinogenicity of Nitrosation Products of Ephedrine, Sarcosine, Folic Acid and Creatinine. *Cancer Res.*, **35**, 1981.